# Evolution of Tembusu Virus in Ducks, Chickens, Geese, Sparrows, and Mosquitoes in Northern China

**DOI:** 10.3390/v10090485

**Published:** 2018-09-10

**Authors:** Guanliu Yu, Yun Lin, Yi Tang, Youxiang Diao

**Affiliations:** 1 College of Animal Science and Technology, Shandong Agricultural University, 61 Daizong Road, Tai’an 271018, Shandong Province, China; yuguanliu@163.com (G.Y.); lyun1994@126.com (Y.L.); 2 Shandong Provincial Key Laboratory of Animal Biotechnology and Disease Control and Prevention, Shandong Agricultural University, 61 Daizong Road, Tai’an 271018, Shandong Province, China; 3 Shandong Provincial Engineering Technology Research Center of Animal Disease Control and Prevention, Shandong Agricultural University, 61 Daizong Road, Tai’an 271018, Shandong Province, China

**Keywords:** Tembusu virus, phylogenetic analysis, duck, protein structure, glycosylation

## Abstract

Tembusu virus (TMUV) is a contagious pathogen from fowl that mainly infects ducks and geese, causing symptoms of high fever, loss of appetite, retarded growth, neurological symptoms, severe duck-drop syndrome, and even death. During an epidemiological investigation of TMUV in Northern China, we isolated 11 TMUV strains from ducks, chickens, geese, sparrows, and mosquitoes (2011–2017). Phylogenetic analysis of the open-reading frames of genes revealed that these strains clustered into Chinese strains II. The nucleotide and amino acid homologies of *NS1* of the strains ranged between 85.8–99.8% and 92.5–99.68%, respectively, which were lower than those of *E* (86.7–99.9% and 96.5–99.9%, respectively), *NS3* (87.6–99.9% and 98.2–99.8%, respectively), and *NS5* (86.5–99.9% and 97.8–99.9%, respectively). Predictions of the tertiary structure of the viral proteins indicated that NS1 in 4 of 11 strains had a protein structure mutation at ^180^TAV^182^ that changed a random crimp into an alpha helix. The protein of 6 of 11 strains had a glycosylation site mutation from NTTD to NITD. Furthermore, epidemiological data suggested that TMUV has been circulating in half of China’s provinces (17 of 34). Our findings, for the first time, have identified the NS1 protein as a potential hypervariable region for genetic evolution. Additionally, the territorial scope of the virus has expanded, requiring strict bio-security measures or a multivalent vaccine to control its spread.

## 1. Introduction

Duck, as a major fowl species in China, offers high-economic benefits with a population of up to 20–30 billion per year [1]. Unfortunately, hemorrhagic ovarian inflammation, which is also named egg-drop syndrome and is mainly caused by Tembusu virus (TMUV), has resulted in large economic losses of several billion dollars for the Chinese duck-rearing industry since April 2010 [2].

TMUV, similar to other flaviviruses, has an approximately 11-kb positive-sense single-stranded RNA genome composed of one long open-reading frame (ORF) encoding three structural proteins, namely capsid, membrane precursor (which is post-translationally cleaved to produce pr and M proteins), and envelope, followed by seven non-structural proteins (NS1, NS2A, NS2B, NS3, NS4A, NS4B, and NS5) [2]. The virus was first isolated from *Culex tritaeniorhynchus* in Malaysia in 1955 [3], after which there were no reports regarding viral outbreaks for more than half a century, excluding a chick-origin TMUV (also named Sitiawan virus) isolated in Malaysia in 2000 [4]. In recent years, after initial outbreaks in Shanghai in April 2010, no major epidemics of duck TMUV (DTMUV) infection were observed throughout 2011, and the disease occurred only sporadically in some individual farms [5]. Nevertheless, the viral infectious disease occurred again in ducks in the major duck-producing region of mainland China in 2012. The cause of these irregular outbreaks demands attention.

As a novel member of *Flavivirus*, this virus has devastating effects on ducks, and can infect geese [6,7,8], chickens [9], mosquitoes [10], sparrows [11], pigeons [12], and mice [13,14]. Furthermore, the virus antibodies have been detected in the serum of 132 duck industry workers, and the serum positive rate was 71.9% (95 of 132) [15]. These findings indicate that the virus has expanded its host range, suggesting it may pose a threat to public health.

Therefore, to better understand the genetic divergence of TMUV in Northern China and provide a foundation for establishing strategies to control TMUV spread, genetic evolution analysis methods (i.e., phylogenetic analysis, predictions of protein tertiary structures and glycosylation sites) were employed to explore the genetic divergence of 11 TMUV strains isolated from ducks, chickens, geese, sparrows, and mosquitoes in Northern China between 2011 and 2017. Furthermore, to objectively reflect TMUV prevalence in China at present, an epidemiological survey of TMUV infection in 17 provinces of China between 2014 and 2017 was also conducted.

## 2. Materials and Methods

### 2.1. Ethics Statement

This study was approved by the Animal Care and Use Committee of Shandong Agricultural University (permit number: 2018061, April, 2018) and performed in accordance with the guidelines of the committee on ethics of experimental animals of Shandong, China.

### 2.2. Epidemiological Survey

In this study, 308 clinical samples (i.e., brains, livers and ovaries of ducks, geese, and chickens) with suspected TMUV infection were collected from 17 provinces between 2014 and 2017. The samples were stored at −80 °C prior to RNA extraction. Total RNA was extracted, treated, tested for quality, stored, and reverse-transcribed to cDNA as described in our previous study [11]. DTMUV-positive samples were examined via reverse transcription-polymerase chain reaction (RT-PCR) using specific primers (Table S2). The PCR program was set as follows: 94 °C for 5 min for the initial denaturation; 33 cycles of denaturation at 94 °C for 45 s, annealing at 57 °C for 55 s, and extension at 72 °C for 50 s; and a final extension at 72 °C for 8 min. The PCR products were purified and cloned into the pMD18-T vector (TaKaRa, Dalian, China), and sequenced using the Sanger sequencing assay (TsingKe Biotech, Beijing, China). Positive results were confirmed by comparing the sequences to reference sequences.

### 2.3. Virus Isolate Descriptions

Over the past 8 years, our team isolated 11 TMUV strains from TMUV-infected ducks, chickens, geese, sparrows, and mosquitoes. As for the virus isolation of ducks, geese, sparrows, and chickens, the liver or brain samples were collected and homogenized in sterile phosphate-buffered saline (PBS, pH 7.2) containing antibiotics (20% *w*/*v*). After centrifugation at 6000 *g* for 30 min, the supernatants were filtered through a 0.2 μm syringe-driven filter (Thermo Scientific, Lenexa, KS, USA). The filtered suspension was then inoculated into 9-day-old SPF duck embryos through the allantoic cavity route [11]. The amnioallantoic fluids of the infected embryos were collected for further passage and RNA extraction. The RNA extraction was carried out with an RNA extraction kit (TIANGEN, Beijing, China), following the manufacturer’s instructions.

With regard to the virus isolation from mosquitoes near to duck farms with TMUV infection outbreaks, the collected mosquitoes were sorted, based on species, with a maximum of 25 adult mosquitoes per pool and stored at −80 °C in 1.5 mL centrifuge tubes [10]. The detection and isolation of mosquito-origin TMUV was the same to that of the above birds-origin TMUV. Nucleotide acid samples of these isolates were sequenced using Sanger sequencing. Detailed information about these isolates is provided in Table 1.

### 2.4. Phylogenetic Analysis

To determine the genetic divergence of the 11 TMUV isolates, 78 TMUV representative ORF sequences were extracted from GenBank. These sequences represented six host species, including 63 ducks, 6 geese, 5 chickens, 2 mosquitoes, 1 sparrow, and 1 pigeon. Detailed information about these strains is listed in Supplementary Table S1.

The phylogenetic trees of the 78 TMUV ORF sequences were constructed via the neighbor-joining method using MEGA software (version 7.0.20, Tempe, AZ, USA) with 1000 bootstrap replicates. Furthermore, the nucleotide and amino acid homologies of the 11 isolates and 67 other representative strains were analyzed using the ClustalW multiple alignment function of BioEdit (version 7.0.5.2, Ibis Biosciences, Carlsbad, CA, USA).

### 2.5. Viral Protein Structure Modeling and Glycosylation Site Prediction

To visually reflect the mutations of the 11 TMUV isolates, the tertiary structures of E, NS1, NS3, and NS5 of these isolates were modeled using Swiss Model (https://swissmodel.expasy.org/interactive). The model templates of E, NS1, NS3, and NS5 proteins were 5wsn.1.A, 5gs6.1A, 2wv9.1, and 4k6m.1, respectively.

The N-glycosylation sites of the four aforementioned proteins were predicted using Glycam (http://glycam.ccrc.uga.edu), which identifies potential N-glycosylation sites by calculating the solvent accessible surface area of Asn in Asn-XThr/Ser/Cys motifs (X represents any amino acid).

## 3. Results

### 3.1. Epidemiological Survey

**Figure 1:**
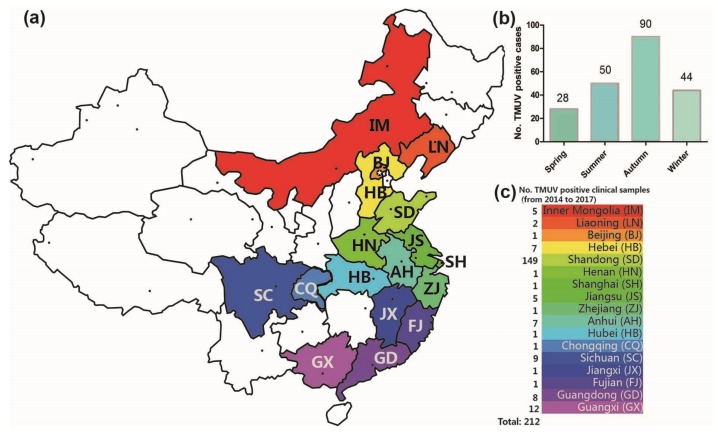
The geographical distribution of clinical samples (**a**). The number of TMUV positive cases in the four seasons (2014–2017) (**b**). In total, 212 Tembusu virus-positive samples were detected via reverse transcription-polymerase chain reaction in 17 Chinese provinces between 2014 and 2017 (**c**).

From 2014 to 2017, 308 clinical samples with suspected TMUV infection were collected in 17 Chinese provinces by our team, and 212 positive samples were identified by RT-PCR and sequence analysis. The TMUV-positive sample spatial distribution information is shown in Figure 1. TMUV has been circulating in half of the provinces of China (17 of 34) (Figure 1a,c). Notably, the incidence in autumn was higher than in spring, summer, and winter (i.e., 90 vs. 28, 50, and 44 respectively; Figure 1b).

### 3.2. Phylogenetic Analysis

**Figure 2:**
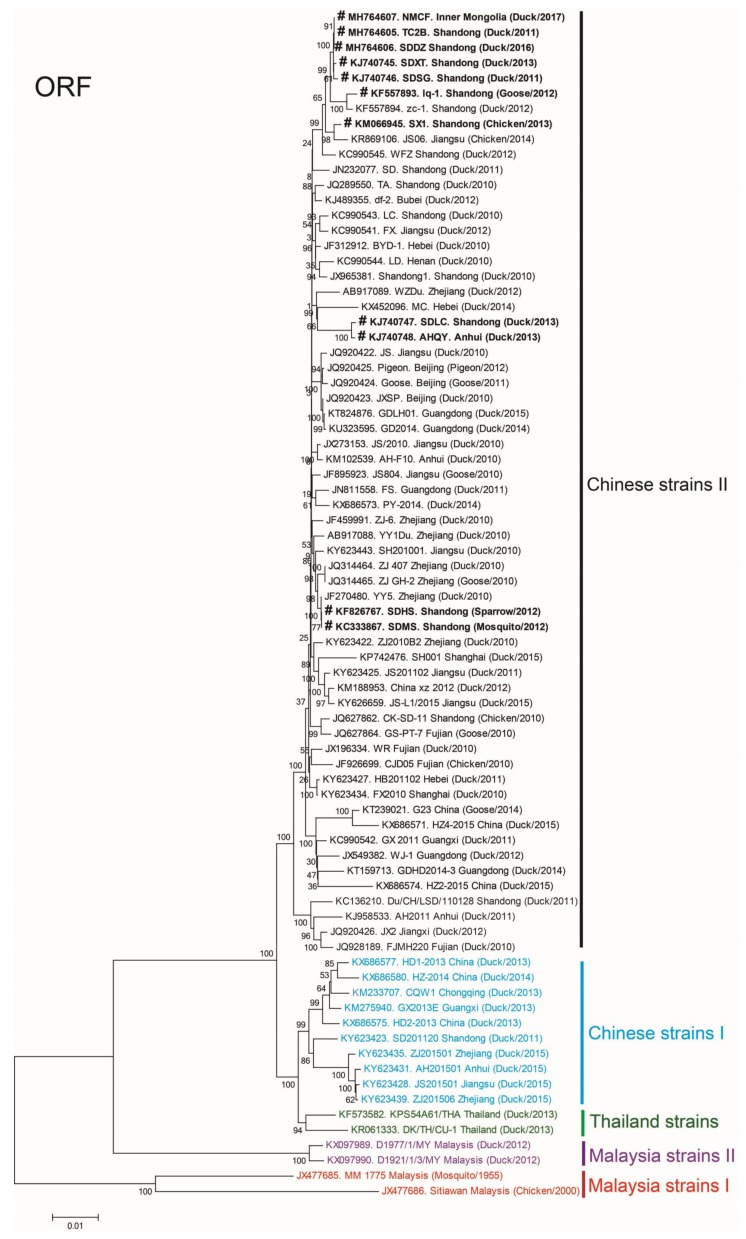
Phylogenetic analysis of the open-reading frame sequences of 78 representative Tembusu virus strains. The phylogenetic trees were constructed using MEGA 7.0 software. Bootstrap consensus values based on 1000 replicates are indicated at each branch point as a percentage. The sequences from the 11 virus strains isolated by in this study are presented in bold. The scale bar indicates the number of nucleotide substitutions per site.

According to phylogenetic analysis of the ORF sequences of 78 representative TMUV strains in South Asia and China, these isolates mainly clustered into five major groups: Malaysia strains I, Malaysia strains II, Thailand strains, Chinese strains I, and Chinese strains II (Figure 2). The phylogenetic trees of the *E*, *NS1*, *NS3*, and *NS5* sequences of the 78 representative strains were analogous to the ORF trees, as presented in Supplementary Figures S1–S4 respectively.

We isolated 11 strains of TMUV from Northern China between 2011 and 2017, and these isolates clustered into Chinese strains II (Figure 2).

Furthermore, based on the nucleotide and amino acid homology analysis of *E*, *NS1*, *NS3*, and *NS5* of the 11 isolates and 67 other representative TMUV strains, the mean nucleotide and amino acid homologies of *NS1* were lower than those of *E*, *NS3*, and *NS5* (i.e., (85.8–99.8% and 92.5–99.68%) vs. (86.7–99.9% and 96.5–99.9%), (87.6–99.9% and 98.2–99.8%), and (86.5–99.9% and 97.8–99.9%) respectively) (Table 2), indicating that NS1 is more variable than the other proteins.

### 3.3. Viral Protein Structure and Glycosylation Site

**Figure 3:**
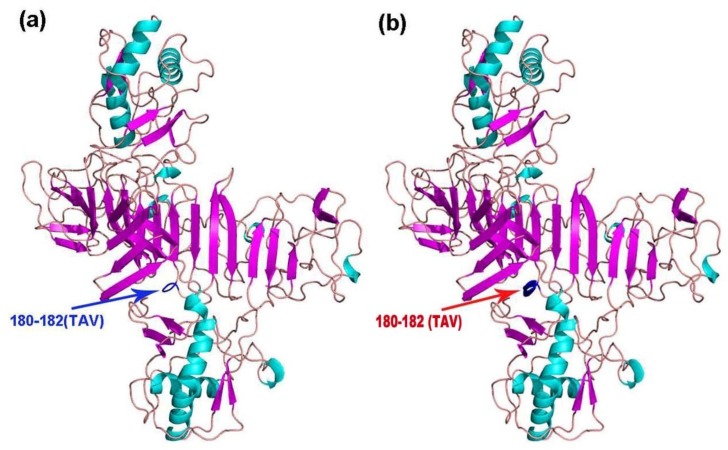
Cartoon scheme view of the NS1 protein structure. (**a**) Conserved structure of NS1 protein; (**b**) mutant NS1 protein structure of KF557893 (1q-1/goose/shandong/2012), KJ740747 (SDLC/duck/shandong/2013), KJ740748 (AHQY/duck/Anhui/2013), and KM066945 (SX1/chicken/shandong/2013).

The tertiary structures of E, NS1, NS3, and NS5 of the 11 TMUV isolates were modeled using Swiss Model. Mutations were only found in NS1 of KF557893 (1q-1/goose/shandong/2012), KJ740747 (SDLC/duck/shandong/2013), KJ740748 (AHQY/duck/Anhui/2013), and KM066945 (SX1/chicken/shandong/2013), as these strains had a ^180^TAV^182^ mutation that changed a random crimp into an alpha helix (Figure 3). However, compared with the conserved sequence, the NS1 amino acid sequences of the mutant strains were unchanged (i.e., TAV vs. TAV).

**Figure 4:**
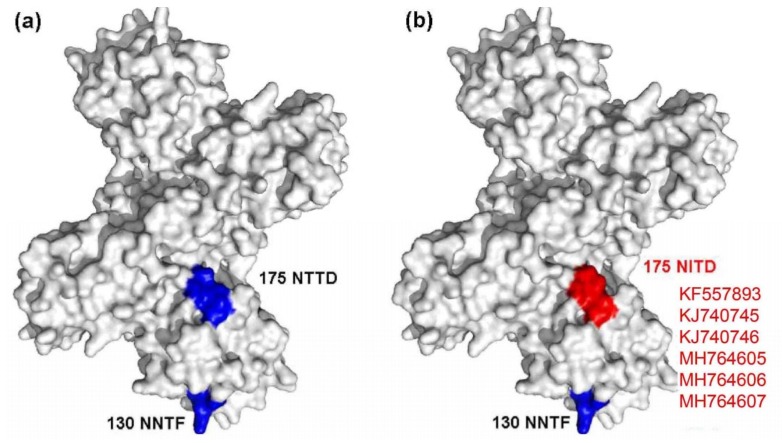
Diagram of NS1 glycosylation sites. (**a**) Conserved glycosylation sites are marked in blue; (**b**) the mutated glycosylation site is marked in red.

A mutated glycosylation site was also found in NS1, and the mutant strains included KF557893 (1q-1/goose/shandong/2012), KJ740745 (SDXT/duck/shandong/2013), KJ740746 (SDSG/duck/shandong/2011), SDDZ/duck/shandong/2016, NMCF/duck/inner mongolia/2017, and TC2B/duck/shandong/2011. Compared with other representative TMUV strains, these strains had a glycosylation site mutation at amino acid site 175 from NTTD to NITD (Figure 4).

## 4. Discussion

DTMUV is a major pathogen that endangers waterfowl, and has caused several billion dollars of losses in the Chinese poultry industry since 2010 [16]. Increasing our knowledge of the genetic evolution of DTMUV is important for realizing the virus protein mutations, and gaining an insight into the protein structure can also provide strategies for vaccine development, which further lay a foundation for controlling the prevalence of DTMUV infection. To date, several TMUV strains have been isolated in China, and their genomic sequences have been uploaded to GenBank; however, few studies have explored the genetic evolution characteristics of these strains’ genomic sequences. No epidemiological surveys of TMUVs have been conducted in China. Thus, based on our previous work (i.e., 11 TMUV strains isolated by our team), the phylogenetic, protein tertiary structure, and glycosylation site analyses were employed to explore the genetic divergence of TMUV in Northern China.

In the present study, according to the phylogenetic trees of *ORF*, *E*, *NS1*, *NS3*, and *NS5* gene sequences of 78 TMUV representative strains, we can see that current Chinese TMUV strains are mainly divided into two distinct genotypes (termed as Chinese strains I and Chinese strains II), and the 11 isolates are mainly clustered into Chinese strains II, which is also named DTMUV II as the dominant strain [5]. Notably, the nucleotide and amino acid homologies of *NS1* were lower than those of *E*, *NS3*, and *NS5* (i.e., (85.8–99.8% and 92.5–99.68%) vs. (86.7–99.9% and 96.5–99.9%), (87.6–99.9% and 98.2–99.8%), and (86.5–99.9% and 97.8–99.9%) respectively), which indicates that NS1 is more variable than the other three proteins.

In the present study, to visually reflect the mutations of the 11 isolates, the tertiary structure predictions of E, NS1, NS3, and NS5 proteins were conducted using Swiss Model. Interestingly, only the NS1 protein (of 4 of 11 strains) had a ^180^TAV^182^ mutation that changed the tertiary structure. Likewise, the N-linked glycosylation site mutation (6 of 11 strains) was also found in the NS1 protein. It is unclear why these mutations occurred only in the NS1 protein. One reason could be the occurrence of “immune selection pressure” [17], which can cause mutations in the structure of a virus’ antigenic proteins in an effort to evade the host immune response. As reported previously, *Flavivirus* NS1 protein is a vital antigenic protein that contains multiple T cell and B cell epitopes that can induce the production of non-neutralizing protective antibodies [18,19,20], and also plays a significant role in immune evasion [21,22,23]. Therefore, these findings indicate that the NS1 protein may be a hypervariable region for genetic evolution.

Furthermore, our epidemiological data revealed that TMUV has been circulating in 17 provinces of China, compared to 9–10 provinces in 2011 [12,24]. This may be because of the expansion of the duck breeding industry and increased transportation capacity of China in recent years, which could accelerate the spread of the virus. Notably, the number of virus host species has increased from four in 2011 (ducks, chickens, geese, and mosquitoes) to six at present (ducks, chickens, geese, sparrows, pigeons, and mosquitoes), which suggests we need to expand the detection range in clinical practice in future. Additionally, reports have indicated that the virus can also infect mice and humans [13,14,15]. These findings imply that the virus has expanded its host range and that it may pose a threat to mammals’ health. Furthermore, the higher incidence in autumn may be associated with the transmission of the virus by mosquitoes [10], the population sizes of which peak in autumn in Northern China.

## 5. Conclusions

In summary, 11 TMUV isolates were obtained from six hosts in Northern China, and all 11 isolates belonged to the dominant cluster in China. The territorial scope of TMUV has expanded in China, and multivalent vaccines are needed to control its spread. Furthermore, our findings indicated that the NS1 protein might be a hypervariable region for genetic evolution. These findings should contribute to the development of strategies for controlling TMUV spread.

## Figures and Tables

**Table 1:** Summary of the 11 Tembusu virus isolates obtained from the northern provinces of China (2011–2017).

Strains	Host	Age	Tissue	Co-Infection	Location	Date	Accession
TC2B	Layer duck	16 d	Liver	-	SD	2011.09.25	MH764605
SDSG	Layer duck	30 d	Liver	H9	SD	2011.11.01	KJ740746
lq-1	Goose	58 d	Brain	GoCV, GPV	SD	2012.10.26	KF557893
SDMS	*Culex* mosquito	-	Body	-	SD	2012.06.01	KC333867 [10]
SDHS	House sparrow	-	Liver	-	SD	2012.12.15	KF826767 [11]
SX1	Layer chicken	45 d	Liver	H9	SD	2013.11.22	KM066945
SDXT	Layer duck	210 d	Liver	DHAV-1	SD	2013.10.01	KJ740745
SDLC	Layer duck	103 d	Liver	H7, H9	SD	2013.11.01	KJ740747
AHQY	Layer duck	160 d	Liver	-	AH	2013.11.06	KJ740748
SDDZ	Meat duck	36 d	Liver	FAdV-4	SD	2016.10.17	MH764606
NMCF	Meat duck	22 d	Liver	DHAV-1	IM	2017.01.02	MH764607

Note, SD: Shandong; AH: Anhui; IM: Inner Mongolia; GoCV: Goose circovirus; GPV: Goose parvovirus; DHAV-1: Duck hepatitis A virus type 1; H7: H7 subtype influenza virus; H9: H9 subtype influenza virus; FAdV-4: Fowl adenovirus serotype 4.

**Table 2:** Nucleotide and amino acid homologies of *E*, *NS1*, *NS3*, and *NS5* of the 11 Tembusu virus isolates (compared with 67 reference strains).

Strains	Nucleotide Homology (nt, %)				Amino Acid Homology (aa, %)			
	E	NS1	NS3	NS5	E	NS1	NS3	NS5
TC2B	86.8–100.0	86.2–100.0	87.7–100.0	86.5–100.0	96.6–100.0	93.5–100.0	98.4–100.0	97.9–100.0
SDSG	86.8–99.9	86.1–99.7	87.6–99.9	86.5–100.0	96.6–100.0	93.5–99.7	98.2–99.8	97.9–100.0
lq-1	86.4–99.6	84.5–99.1	87.5–100.0	86.3–99.5	95.6–99.4	88.4–98.3	98.1–100.0	97.1–99.4
SDMS	87.0–100.0	86.1–100.0	87.8–100.0	86.8–100.0	96.8–100.0	92.9–100.0	98.4–100.0	97.9–100.0
SDHS	87.0–100.0	86.1–100.0	87.8–100.0	86.8–100.0	96.8–100.0	92.9–100.0	98.4–100.0	97.9–100.0
SX1	86.6–99.7	85.0–99.6	87.4–99.7	86.3–99.8	96.2–99.6	91.5–99.4	97.6–99.2	97.8–99.9
SDXT	86.6–99.9	86.4–99.7	87.8–99.9	86.5–99.9	96.4–99.8	93.8–99.7	98.4–100.0	97.9–100.0
SDLC	86.5–100.0	85.4–99.8	87.3–99.6	86.2–99.9	96.8–100.0	92.0–99.7	97.6–99.2	98.0–100.0
AHQY	86.5–100.0	85.2–99.8	87.5–99.6	86.2–99.9	96.8–100.0	91.8–99.7	98.2–99.5	97.9–99.9
SDDZ	86.8–100.0	86.2–100.0	87.7–100	86.5–100.0	96.6–100.0	93.5–100.0	98.4–100.0	97.9–100.0
NMCF	86.8–100.0	86.2–100.0	87.7–100	86.5–100.0	96.6–100.0	93.5–100.0	98.4–100.0	97.9–100.0
Mean value	86.7–99.9	85.8–99.8	87.6–99.9	86.5–99.9	96.5–99.9	92.5–99.68	98.2–99.8	97.8–99.9

## Supplementary Materials

The following are available online at http://www.mdpi.com/1999-4915/10/9/485/s1, Figure S1: Phylogenetic analysis of E gene sequences of 78 representative Tembusu virus strains. Figure S2: Phylogenetic analysis of NS1 gene sequences of 78 representative Tembusu virus strains. Figure S3: Phylogenetic analysis of NS3 gene sequences of 78 representative Tembusu virus strains. Figure S4: Phylogenetic analysis of NS5 gene sequences of 78 representative Tembusu virus strains. Table S1: Description of 78 TMUV representative isolates used in this study. Table S2: Primers used in the study.
