# Supplementary figures and images for: Evolution of Tembusu Virus in Ducks, Chickens, Geese, Sparrows, and Mosquitoes in Northern China
Source: Viruses. 2018 Sep 10;10(9):485. doi: 10.3390/v10090485 (PMC6164154; doi:10.3390/v10090485)

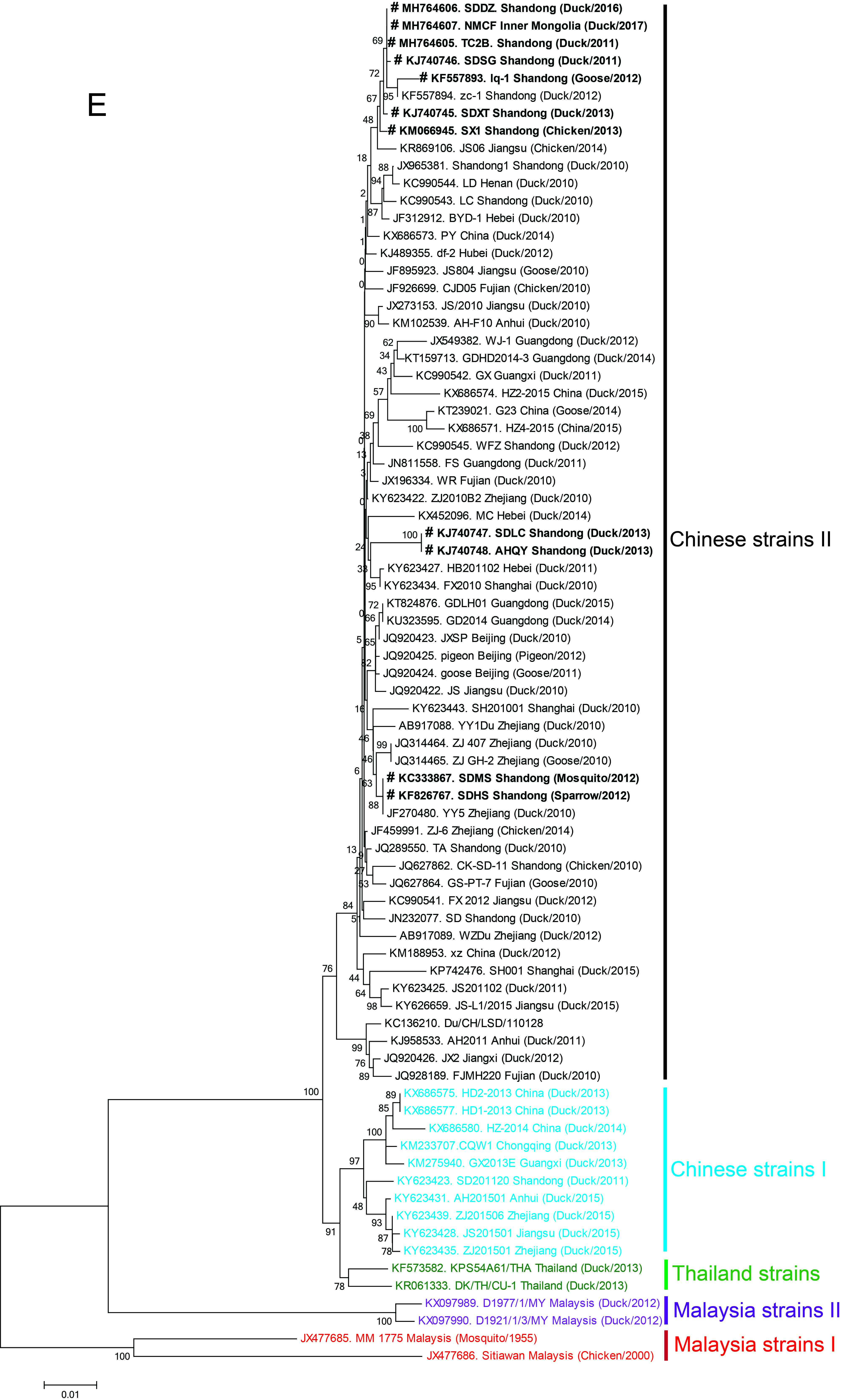

Supplement: Supplementary file 1 [file viruses-10-00485-s001.zip › Supplementary materials/Figure S1.jpg]

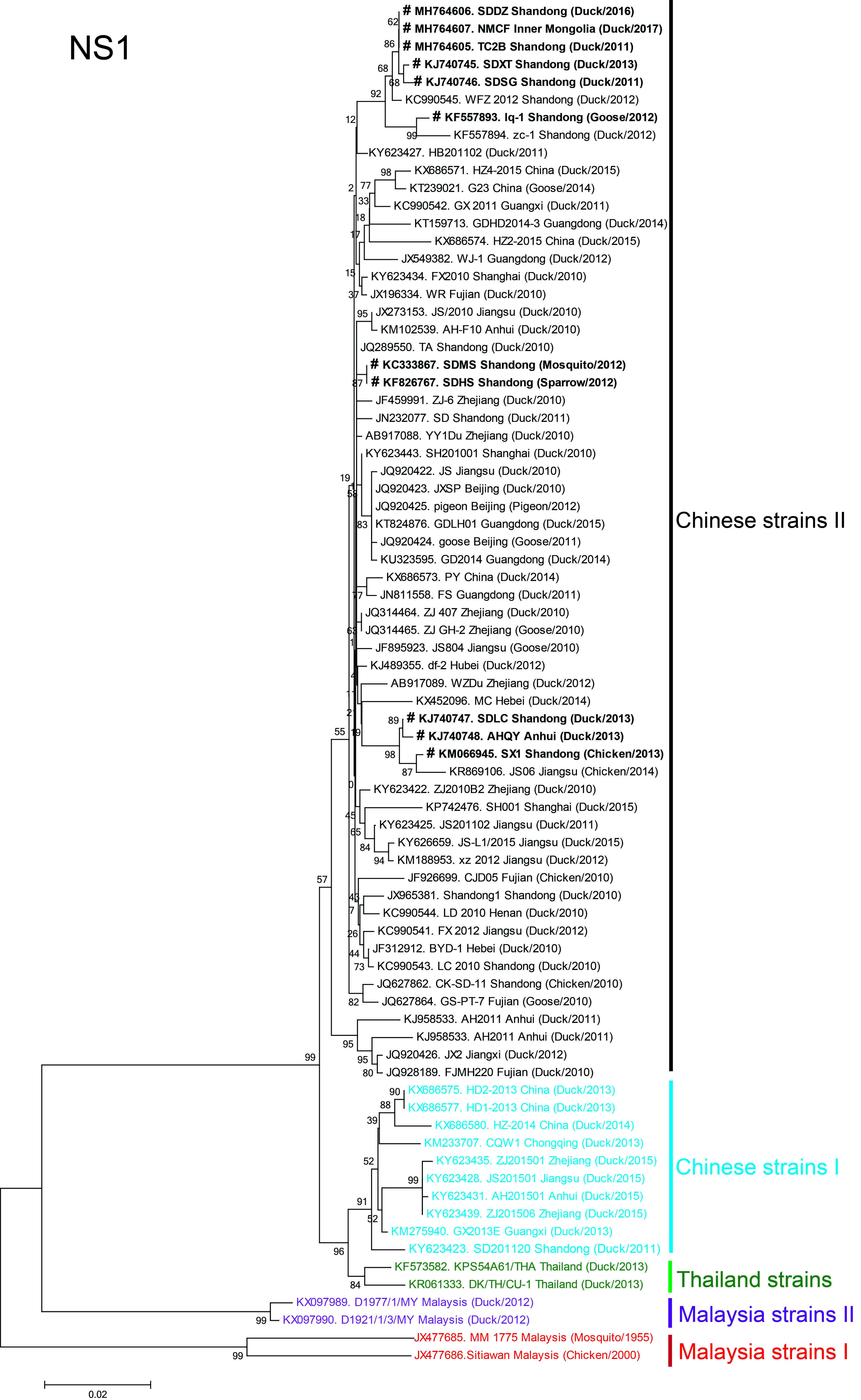

Supplement: Supplementary file 1 [file viruses-10-00485-s001.zip › Supplementary materials/Figure S2.jpg]

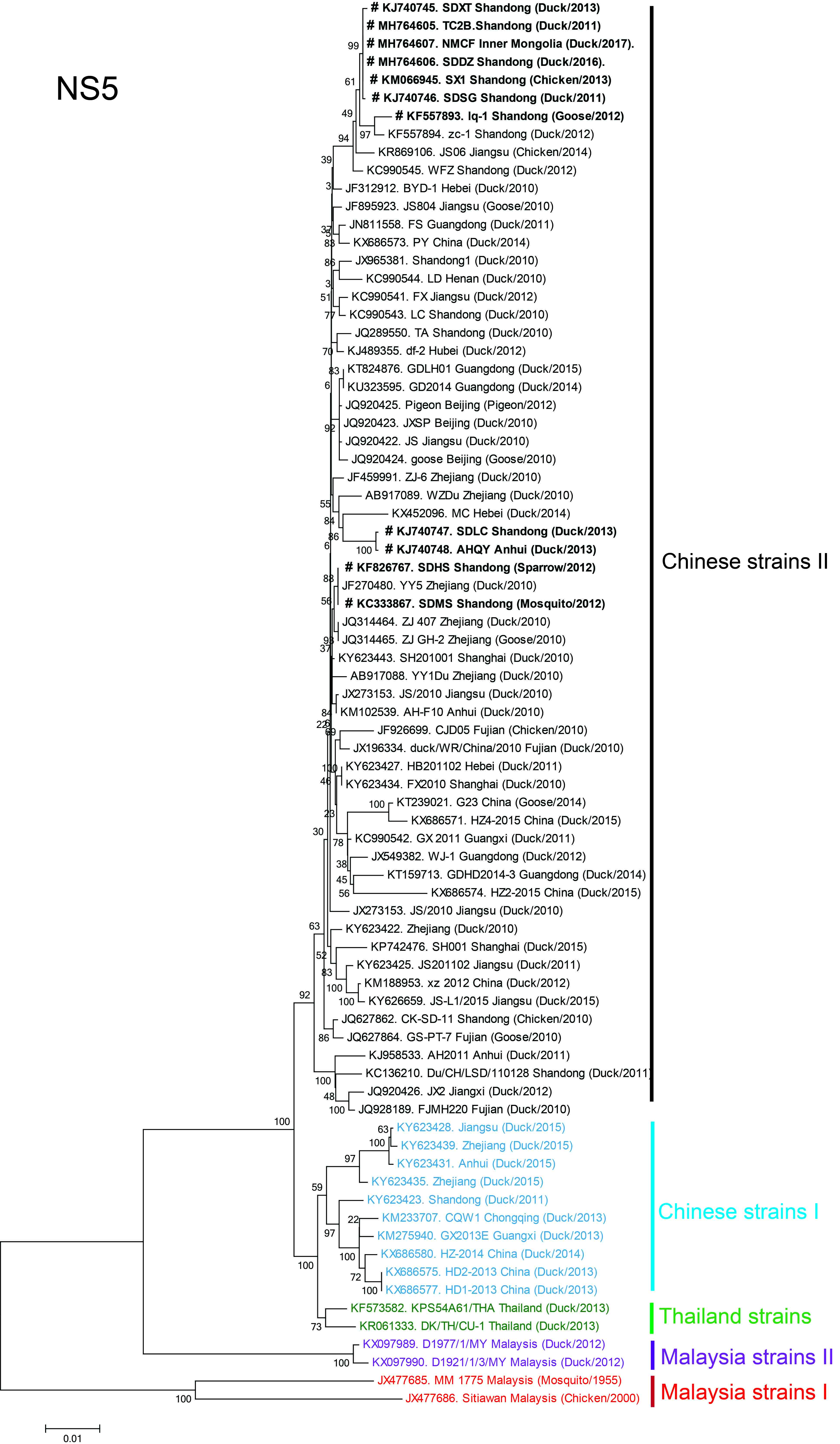

Supplement: Supplementary file 1 [file viruses-10-00485-s001.zip › Supplementary materials/Figure S4.jpg]
