# Supplementary material for: Evolution of Tembusu Virus in Ducks, Chickens, Geese, Sparrows, and Mosquitoes in Northern China
Source: Viruses. 2018 Sep 10;10(9):485. doi: 10.3390/v10090485 (PMC6164154; doi:10.3390/v10090485)
Supplement: Supplementary file 1 [file viruses-10-00485-s001.zip › Supplementary materials/Figure S1, S2, S3, and S4.doc]

**Figure S1.** Phylogenetic analysis of E gene sequences of 78 representative Tembusu virus strains. The phylogenetic trees were constructed using MEGA 7.0 software. Bootstrap consensus values based on 1000 replicates are indicated at each branch point as a percentage. # indicates the sequences from the virus isolated in this study. The scale bar indicates the number of nucleotide substitutions per site.

**Figure S2.** Phylogenetic analysis of NS1 gene sequences of 78 representative Tembusu virus strains. The phylogenetic trees were constructed using MEGA 7.0 software. Bootstrap consensus values based on 1000 replicates are indicated at each branch point as a percentage. # indicates the sequences from the virus isolated in this study. The scale bar indicates the number of nucleotide substitutions per site.

**Figure S3.** Phylogenetic analysis of NS3 gene sequences of 78 representative Tembusu virus strains. The phylogenetic trees were constructed using MEGA 7.0 software. Bootstrap consensus values based on 1000 replicates are indicated at each branch point as a percentage. # indicates the sequences from the virus isolated in this study. The scale bar indicates the number of nucleotide substitutions per site.

**Figure S4.** Phylogenetic analysis of NS5 gene sequences of 78 representative Tembusu virus strains. The phylogenetic trees were constructed using MEGA 7.0 software. Bootstrap consensus values based on 1000 replicates are indicated at each branch point as a percentage. # indicates the sequences from the virus isolated in this study. The scale bar indicates the number of nucleotide substitutions per site.
