# Supplementary material for: Evolution of Tembusu Virus in Ducks, Chickens, Geese, Sparrows, and Mosquitoes in Northern China
Source: Viruses. 2018 Sep 10;10(9):485. doi: 10.3390/v10090485 (PMC6164154; doi:10.3390/v10090485)
Supplement: Supplementary file 1 [file viruses-10-00485-s001.zip › Supplementary materials/Table S1 and S2.doc]

**Table S1.** Description of 78 TMUV representative isolates used in this study.

| **Strain** | **Access number** | **Host** | **Date** | **Location** |
| --- | --- | --- | --- | --- |
| MM1775 | JX477685 | Mosquito | 1955 | Malaysia |
| Sitiawan virus | JX477686 | Chicken | 2000 | Malaysia |
| TC2B | MH764605 | Duck | 2010 | Shandong |
| YY1Du | AB917088 | Duck | 2010 | Zhejiang |
| YY5 | JF270480 | Duck | 2010 | Zhejiang |
| BYD-1 | JF312912 | Duck | 2010 | Hebei |
| ZJ-6 | JF459991 | Duck | 2010 | Zhejiang |
| JS804 | JF895923 | Geese | 2010 | Jiangsu |
| CJD05 | JF926699 | Chicken | 2010 | Fujian |
| TA | JQ289550 | Duck | 2010 | Shandong |
| ZJ 407 | JQ314464 | Duck | 2010 | Zhejiang |
| ZJ GH-2 | JQ314465 | Geese | 2010 | Zhejiang |
| CK-SD-11 | JQ627862 | Chicken | 2010 | Shandong |
| GS-PT-7 | JQ627864 | Geese | 2010 | Fujian |
| FJMH220 | JQ928189 | Duck | 2010 | Fujian |
| duck/WR/China/2010 | JX196334 | Duck | 2010 | Fujian |
| JS/2010 | JX273153 | Duck | 2010 | Jiangsu |
| Shandong1 | JX965381 | Duck | 2010 | Shandong |
| LC_2010 | KC990543 | Duck | 2010 | Shandong |
| LD_2010 | KC990544 | Duck | 2010 | Henan |
| AH-F10 | KM102539 | Duck | 2010 | Anhui |
| ZJ2010B2 | KY623422 | Duck | 2010 | Zhejiang |
| FX2010 | KY623434 | Duck | 2010 | Shanghai |
| SH201001 | KY623443 | Duck | 2010 | Shanghai |
| SD | JN232077 | Duck | 2011 | Shandong |
| FS | JN811558 | Duck | 2011 | Guangdong |
| Du/CH/LSD/110128 | KC136210 | Duck | 2011 | Shandong |
| GX_2011 | KC990542 | Duck | 2011 | Guangxi |
| SDSG | KJ740746 | Duck | 2011 | Shandong |
| AH2011 | KJ958533 | Duck | 2011 | Anhui |
| SD201120 | KY623423 | Duck | 2011 | Shandong |
| JS201102 | KY623425 | Duck | 2011 | Jiangsu |
| HB201102 | KY623427 | Duck | 2011 | Hebei |
| WZDu | AB917089 | Duck | 2012 | Zhejiang |
| pigeon | JQ920425 | pigeon | 2012 | Beijing |
| JX2 | JQ920426 | Duck | 2012 | Jiangxi |
| WJ-1 | JX549382 | Duck | 2012 | Guangdong |
| SDMS | KC333867 | Mosquito | 2012 | Shandong |
| FX_2012 | KC990541 | Duck | 2012 | Jiangsu |
| WFZ_2012 | KC990545 | Duck | 2012 | Shandong |
| lq-1 | KF557893 | Geese | 2012 | Shandong |
| zc-1 | KF557894 | Duck | 2012 | Shandong |
| SDHS | KF826767 | Sparrow | 2012 | Shandong |
| df-2 | KJ489355 | Duck | 2012 | Hubei |
| xz 2012 | KM188953 | Duck | 2012 | Jiangsu |
| D1977/1/MY | KX097989 | Duck | 2012 | Malaysia |
| D1921/1/3/MY | KX097990 | Duck | 2012 | Malaysia |
| KPS54A61/THA | KF573582 | Duck | 2013 | Thailand |
| SDXT | KJ740745 | Duck | 2013 | Shandong |
| SDLC | KJ740747 | Duck | 2013 | Shandong |
| AHQY | KJ740748 | Duck | 2013 | Anhui |
| SX1 | KM066945 | Chicken | 2013 | Shandong |
| CQW1 | KM233707 | Duck | 2013 | Chongqing |
| GX2013E | KM275940 | Duck | 2013 | Guangxi |
| DK/TH/CU-1 | KR061333 | Duck | 2013 | Thailand |
| HD2-2013 | KX686575 | Duck | 2013 | China |
| HD1-2013 | KX686577 | Duck | 2013 | China |
| JS06 | KR869106 | Chicken | 2014 | Jiangsu |
| GDHD2014-3 | KT159713 | Duck | 2014 | Guangdong |
| G23 | KT239021 | Geese | 2014 | Guangdong |
| GD2014 | KU323595 | Duck | 2014 | Guangdong |
| MC | KX452096 | Duck | 2014 | Hebei |
| PY-2014 | KX686573 | Duck | 2014 | China |
| HZ-2014 | KX686580 | Duck | 2014 | China |
| SH001 | KP742476 | Duck | 2015 | Shanghai |
| GDLH01 | KT824876 | Duck | 2015 | Guangdong |
| HZ4-2015 | KX686571 | Duck | 2015 | China |
| HZ2-2015 | KX686574 | Duck | 2015 | China |
| JS201501 | KY623428 | Duck | 2015 | Jiangsu |
| AH201501 | KY623431 | Duck | 2015 | Anhui |
| ZJ201501 | KY623435 | Duck | 2015 | Zhejiang |
| ZJ201506 | KY623439 | Duck | 2015 | Zhejiang |
| JS-L1/2015 | KY626659 | Duck | 2015 | Jiangsu |
| SDDZ | MH764606 | Duck | 2016 | Shandong |
| NMCF | MH764607 | Duck | 2017 | Inner Mongolia |
| JS | JQ920422 | Duck | */* | Jiangsu |
| JXSP | JQ920423 | Duck | */* | Beijing |
| goose | JQ920424 | Geese | */* | Beijing |

**Table S2.** Primers used in the study.

| **Primer name** | **Sequences (5′ - 3′)** | **Size (bp)** | **Purpose** |
| --- | --- | --- | --- |
| TMUV | F:CTGAAATAGCGGAAGCACTGA | 277 | RT-PCR for clinical sample detection |
|  | R:CTGGAGGTGTGGCTGTCAT |  |  |
| TMUV-E | F:TTCAGCTGTCTGGGGATGCA | 1503 | E gene amplification |
|  | R:GGCATTGACATTTACTGCCA |  |  |
| TMUV-NS1 | F:GACACGGGGTGCTCAATCGACTT | 1056 | NS1 gene amplification |
|  | R:AGCCATGACCTTTGATTTGAT |  |  |
| TMUV-NS3 | F:GGAGGAGTCATCTGGGATGTG | 1857 | NS3 gene amplification |
|  | R:TCTCTTTCCACTCGCAAAATC |  |  |
| TMUV-NS5 | F:GAACTGGCAGAACTTTGGGGGAG | 2711 | NS5 gene amplification |
|  | R:TTACAAGACACCTTCACTCCAGC |  |  |
